# 5‐Fluorouracil reduces the fibrotic scar via inhibiting matrix metalloproteinase 9 and stabilizing microtubules after spinal cord injury

**DOI:** 10.1111/cns.13930

**Published:** 2022-08-02

**Authors:** Yang Xu, Xiuying He, Yangyang Wang, Jiao Jian, Xia Peng, Lie Zhou, Yi Kang, Tinghua Wang

**Affiliations:** ^1^ Institute of Neurological Disease, West China Hospital, Sichuan University & The Research Units of West China Chinese Academy of Medical Sciences Chengdu China; ^2^ Laboratory of Anesthesia and Critical Care Medicine, Department of Anesthesiology, Translational Neuroscience Center, West China Hospital Sichuan University Chengdu China; ^3^ Institute of Neuroscience, Laboratory Zoology Department Kunming Medical University Kunming China; ^4^ Yunnan Key Laboratory of Stem Cell and Regenerative Medicine, Biomedical Engineering Research Center Kunming Medical University Kunming China; ^5^ National‐Local Joint Engineering Research Center of Translational Medicine of Anesthesiology, West China Hospital Sichuan University Chengdu China

**Keywords:** 5‐FU, fibrotic scar, hemisection SCI, MMP9

## Abstract

**Aims:**

Fibrotic scars composed of a dense extracellular matrix are the major obstacles for axonal regeneration. Previous studies have reported that antitumor drugs promote neurofunctional recovery.

**Methods:**

We investigated the effects of 5‐fluorouracil (5‐FU), a classical antitumor drug with a high therapeutic index, on fibrotic scar formation, axonal regeneration, and functional recovery after spinal cord injury (SCI).

**Results:**

5‐FU administration after hemisection SCI improved hind limb sensorimotor function of the ipsilateral hind paws. 5‐FU application also significantly reduced the fibrotic scar formation labeled with aggrecan and fibronectin‐positive components, Iba1^+^/CD11b^+^ macrophages/microglia, vimentin, chondroitin sulfate proteoglycan 4 (NG2/CSPG4), and platelet‐derived growth factor receptor beta (PDGFRβ)^+^ pericytes. Moreover, 5‐FU treatment promoted stromal cells apoptosis and inhibited fibroblast proliferation and migration by abrogating the polarity of these cells and reducing matrix metalloproteinase 9 expression and promoted axonal growth of spinal neurons via the neuron‐specific protein doublecortin‐like kinase 1 (DCLK1). Therefore, 5‐FU administration impedes the formation of fibrotic scars and promotes axonal regeneration to further restore sensorimotor function after SCI.

## Graphical abstract

5‐FU treatment inhibited fibroblast proliferation and migration by abrogating the polarity of these cells and reducing matrix metalloproteinase 9 expression and further promoted axonal regeneration to restore sensorimotor function after SCI.

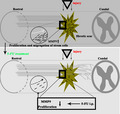

## 1 INTRODUCTION

Spinal cord injury (SCI) is one of the most serious neurological diseases caused by traumatic injury,^1^ with a high incidence and disability rate.^2^ There are currently no effective therapeutic strategies to improve loss of function in patients with SCI.^3^, ^4^

Scar formation after SCI is an important factor that inhibits regeneration of the nerve axon, resulting in permanent functional deficits.^5^, ^6^ Physical and chemical barriers are generated to prevent axons from passing through the injured site and entering the scar free spinal region.^7^ In mammals, inhibition of scar formation can promote axon regeneration at the lesion site within the central nervous system.^8^ Resident astrocytes are progressively activated and become hypertrophic, eventually forming a glial scar.^9^ Non‐neural cells (e.g., NG2 macrophages, meningeal and/or vascular‐derived fibroblasts, pericytes, ependymal cells, and phagocytic macrophages) from the periphery form a lesion core (also known as called fibrotic scar) through the damaged blood brain barrier or other spaces. This group of cells was collectively called stromal cells.^10^, ^11^ During cell aggregation, dense extracellular matrix molecules (ECMMs), including inhibitory ECMs (i.e., fibronectin, aggrecan, and versican), are secreted.^10^ These inhibitory molecules were identified as the major impediments for axonal regeneration. Detection of these components can indirectly reflect the severity of SCI.^12^

Recent studies have shown that antitumor drugs, especially anti‐cell cycle drugs, have achieved positive effects in the treatment of SCI. The antitumor drug paclitaxel can enhance axonal regeneration and reduce scar formation after SCI.^13^ However, paclitaxel cannot cross the blood–brain barrier, which restricts its application for the treatment of neurological disorders. In contrast to paclitaxel, epothilone can easily penetrate the blood–brain barrier and has comparable biological effects.^14^ Other antitumor drugs, such as 5‐FU, a pyrimidine analog that inhibits the cell cycle with a wide range of antitumor activities, are blood brain barrier permeable.^15^ 5‐FU has been used as a routine therapeutic drug in the systemic treatment of hepatocellular carcinoma.^16^ However, few studies have reported the effect of 5‐FU on ameliorating SCI.

Here, we investigated the effects of 5‐FU in a rat hemisection SCI model. We observed that systemic 5‐FU treatment for 4 consecutive days resulted in reduced fibrotic scar formation and improved locomotor function after spinal cord hemisection injury. The current study provides evidence for the use of 5‐FU as a novel therapeutic for the clinical treatment of SCI.

## 2 METHODS

### 2.1 Animal grouping

Animal feeding and care were conducted in strict compliance with the Chinese Experimental Animal Protection and Ethics Committee and guidelines for the care and use of laboratory animals published by the National Institutes of Health. All surgical interventions were ethical approved by the “sichuan provincial committee for experimental animal management”. Animal data reporting has followed the ARRIVE guidelines.^17^

Adult female Sprague Dawley (SD) rats were divided into four groups as follows: Naïve group (*n* = 6), sham group (rats that were subjected to partial laminectomy without SCI, *n* = 6), vehicle group (SCI rats that received saline injection, *n* = 10/timepoint, total *N* = 50), and 5‐FU group (SCI rats that received 5‐FU treatment, *n* = 10/timepoint, total *N* = 50). The 5‐FU group was exposed to intraperitoneal (i.p) injections of 5‐FU (5 mg/kg) immediately for 4 consecutive days, and the same dose of normal saline was administered to the vehicle group.

### 2.2 Scar area quantification

Ten‐millimeter sections centered on the lesion were dissected from the spinal cord and the area of brown discoloration was quantified as a measurement of scar area. For the assessment of the fibrotic scar area, glial fibrillary acidic protein (GFAP) negative regions were measured surrounded by dense GFAP‐positive staining. The direct measurements of the fibrotic scar were performed by quantification of aggrecan, fibronectin, PDGFRβ, and vimentin positive staining. To quantify all of these immunostains mentioned above, at least six coronal sections per animal were randomly selected and immunostained for data statistics.

Methods of Animal model establishment, Basso Beattie Bresnahan (BBB) scale, horizontal ladder‐walking test, thermal hyperalgesia test, mechanical hyperalgesia test, tissue harvest, total white blood cell (WBC) counts, flow cytometry, liquid chromatography tandem mass spectrometry (LC–MS/ MS), Nissl staining, Apoptosis, cell culture, wound healing assay, Immunofluorescence assay, Western blots, and statistics analysis were mentioned as Supplementary methods in the Supplementary information.

## 3 RESULTS

### 3.1 5‐FU promotes sensorimotor function recovery after SCI

**FIGURE 1:**
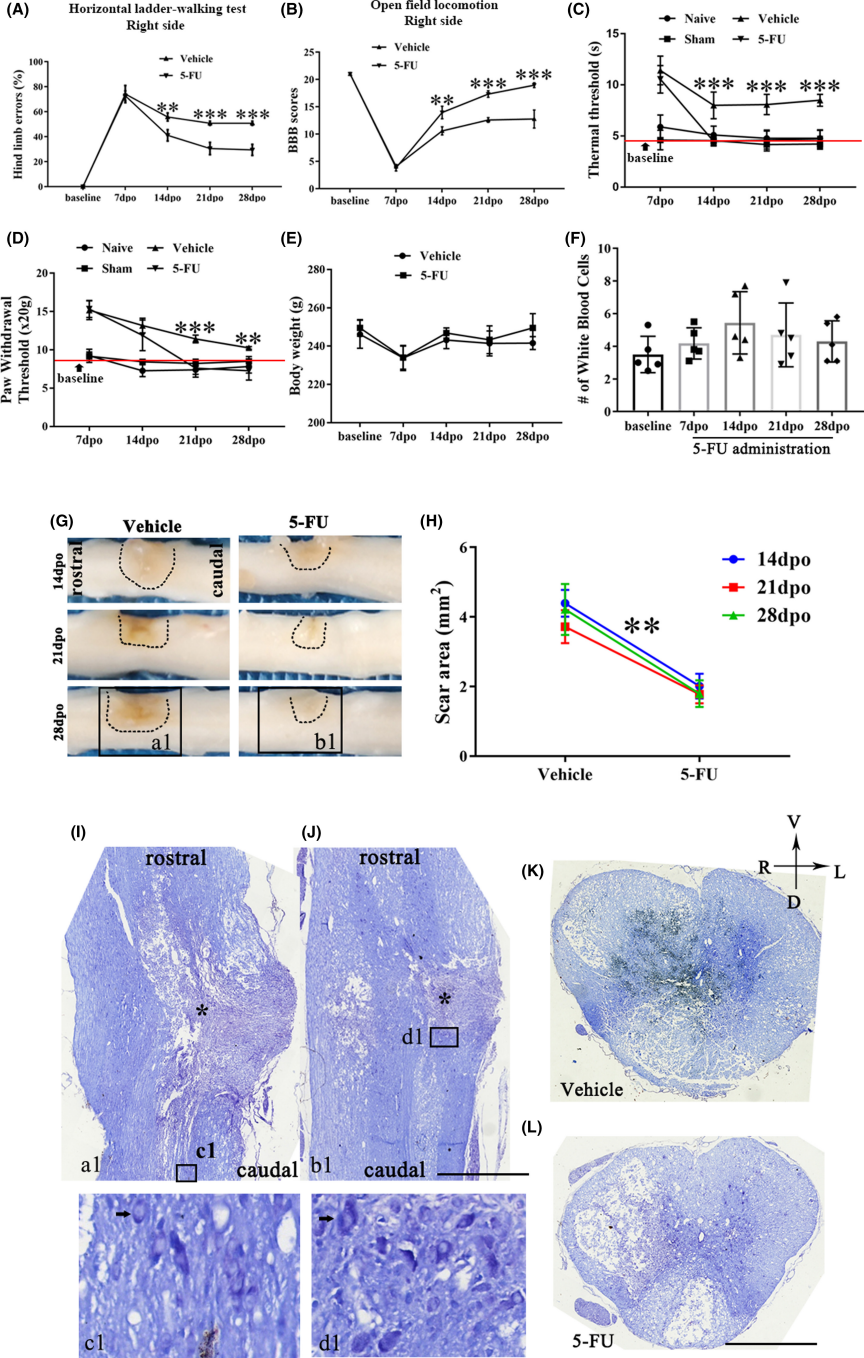
5‐FU administration promotes sensorimotor functional recovery and reduces the scar formation after SCI. (A) Percentage of hind limb errors in the horizontal ladder test after SCI (*n* = 6/group). (B) BBB scores of SCI rats with different approaches (*n* = 6/group). (C–D) Statistical results of thermal and paw withdrawal thresholds in SCI rats after different treatments (*n* = 6/group). (E) Body weight of SCI rats with different approaches at 7‐, 14‐, 21‐ and 28‐days post‐operation (*n* = 6/group). (F) Total WBC counts in the blood of 5‐FU treated group at different time points (*n* = 5/group). (G) Low power photographs of the spinal cord between vehicle and 5‐FU groups. (H) Scar area of the lesion site (*n* = 6). (I–J) Nissl's staining of the coronal spinal cord in Figure a1 and b1 form Figure G. Nissl body in the cytoplasm of nerve cells (dark blue‐purple, black arrow). * indicates the lesion core. The bottom panels are an enlargement of the box in Figure I/J. (K–L) Nissl's staining of the spinal cross section in the asterisked central plane. Graphical data are presented as the mean ± standard deviation. Scale bar = 1 mm. R: right, L: left, V: ventral, D: dorsal. *p < 0.05, **p < 0.01, ***p < 0.001

There was a significantly increase in hind limb faults made by the ipsilateral hindlimb 7 days post operation (dpo) compared with the baseline (Figure 1A). Compared with the vehicle group, 5‐FU treatment significantly reduced the hind limb error rate of the ipsilateral hind paws, the effects of which started on 14dpo and lasted until the final detection day (Figure 1A). Similarly, the BBB scores of the hindlimb ipsilateral to the injury site were also significantly elevated in the 5‐FU‐treated group compared to that of vehicle group (Figure 1B). The thermal thresholds in all hemisection models were significantly higher than that in the sham group and naïve rats from 7dpo (Figure 1C). After 5‐FU administration, the thermal threshold was markedly decreased and returned to baseline levels relative to the vehicle group, the effects of which started on 14dpo (Figure 1C). In the paw withdrawal threshold data, 5‐FU‐treated animals showed decreased latencies for withdrawal, starting at 21dpo (Figure 1D). The intraperitoneal (i.p.) injected dose was well tolerated, with no obvious reduction in the animal's weight and WBC counts (*p* > 0.05, Figure 1E,F). Besides, significant increase of serum alanine aminotransferase (ALT), alkaline phosphatase (AST), creatinine (CREA), and urea were not observed in rats after 5‐FU administration (*p* > 0.05, see Figure S1).

### 3.2 5‐FU administration reduces scar formation and promotes the recovery of neuronal viability near the epicenter

Data showed that the scar area was significantly reduced in 5‐FU‐treated rats compared with that in the vehicle group, starting at 14dpo (*p* < 0.01, Figure 1G,H). In addition, the cytoarchitectonic organization of the injured spinal cord was visualized using Nissl staining of the coronal spinal cord (Figure a1,b1 in Figure 1G), which revealed that neurons in the vehicle group showed pyknosis, accompanied by collapse and karyolysis of the Nissl body, unlike in the 5‐FU group (Figure 1I,J). The maximum diameter of the remaining neuro‐soma, near the scar of the gray matter, was greater than that of vehicle group, after 5‐FU administration (Figure 1c1,d1). The gray matter in the spinal cord of vehicle rats was disintegrated and necrotic. There was less cystic degeneration in the 5‐FU models than in the vehicle rats (Figure 1K,L).

### 3.3 5‐FU administration attenuates the fibrotic scar formation

**FIGURE 2:**
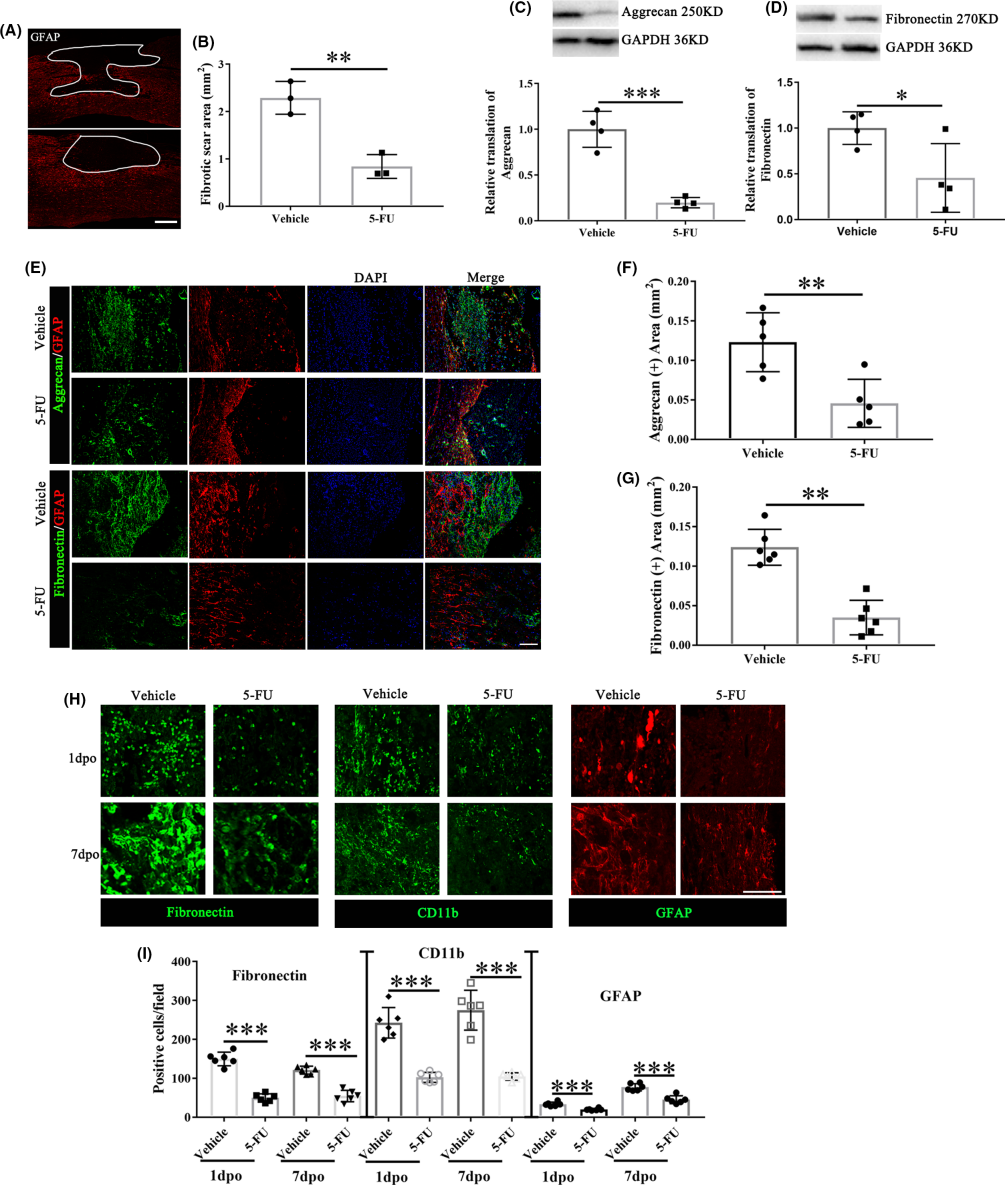
Fibrotic scar areas and local distribution of fibroblasts and glial cells between vehicle and 5‐FU groups. (A) Immunohistochemistry of rat coronal spinal sections at 28dpo with anti‐GFAP antibody. White line area indicated the fibrotic scar areas. Scale bar = 200 μm. (B) Quantification of the fibrotic scar area (*n* = 3/group). (C) Expression levels of aggrecan in the injured spinal cord of vehicle and 5‐FU treated animals (*n* = 4/group). (D) Expression levels of fibronectin in the injured spinal cord of vehicle and 5‐FU treated animals (*n* = 4/group). (E) Immunohistochemistry of rat coronal spinal sections at 28dpo with anti‐GFAP, anti‐aggrecan and anti‐fibronectin antibodies between vehicle and 5‐FU groups. Scale bar = 50 μm. (F–G) Quantification of the aggrecan and fibronectin positive area (*n* = 6/group). (H) Immunohistochemistry of the epicenter at 1dpo and 7dpo with anti‐fibronectin, anti‐CD11b and anti‐GFAP antibodies between vehicle and 5‐FU groups. Scale bar = 50 μm. (I) Quantification of the fibronectin, CD11b and GFAP positive cells at 1dpo and 7dpo (*n* = 6/group). DAPI (blue) labeled cell nucleus. Graphical data are presented as the mean ± standard deviation. *p < 0.05, **p < 0.01, ***p < 0.001

**FIGURE 3:**
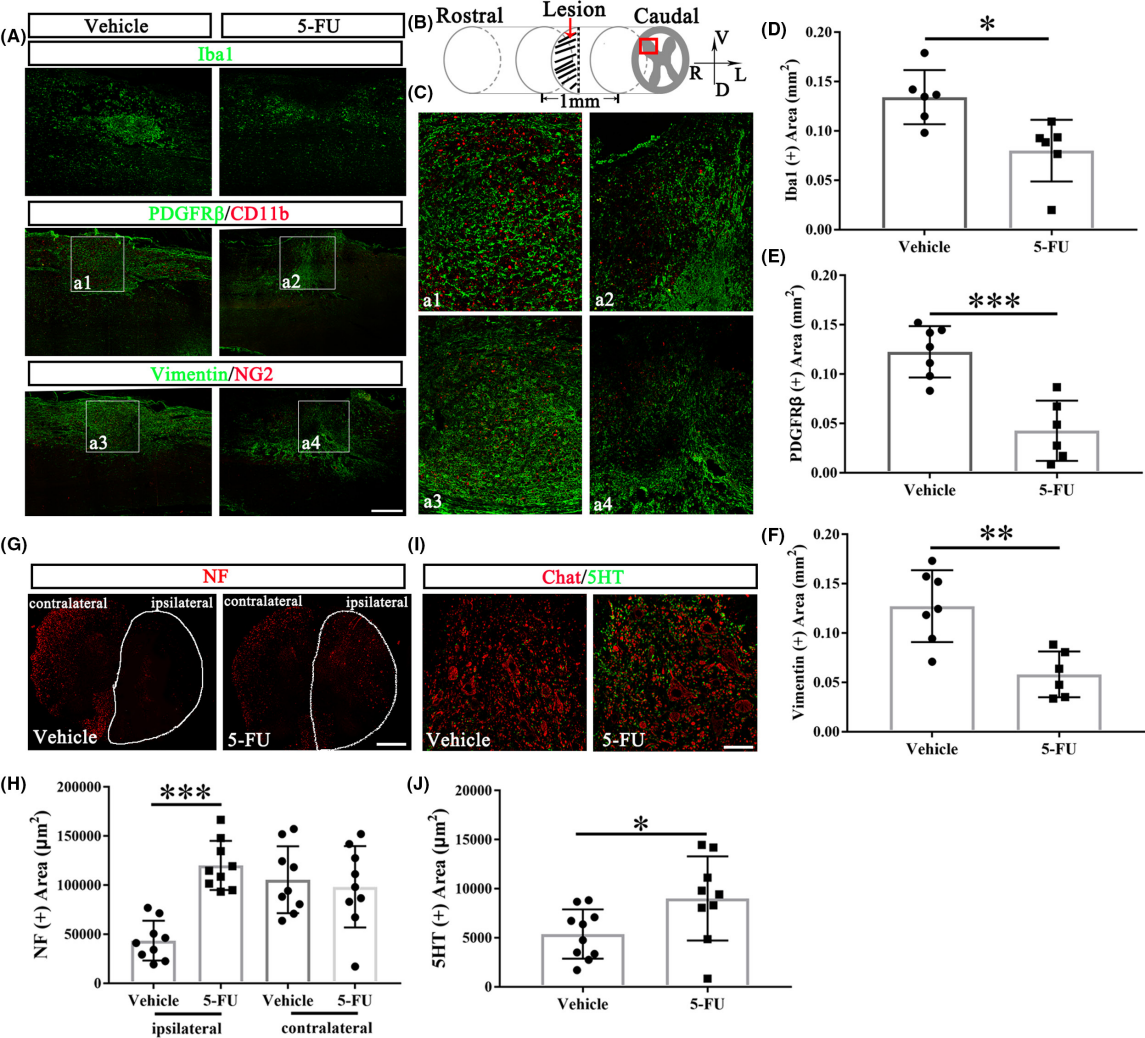
5‐FU administration attenuates fibrotic scar formation. (A) Immunohistochemistry of rat coronal spinal sections at 28dpo. Scale bar = 200 μm. (B) Schematic representation of the lesion and displayed regions (red box) analyzed by Image J to determine fibrotic scar formation (Figure A–F) and axonal regeneration/preservation (Figure 3G–J). (C) The enlargements of a1‐a4 from Figure A. (D–F) Quantification of expression areas of Iba1, PDGFRβ and vimentin (*n* = 6/group). (G) Immunohistochemistry cross section of rat spinal cord at 28dpo with anti‐NF antibody staining. White line area indicates the ipsilateral spinal cord. Scale bar = 100 μm. (H) Quantification of the NF positive area (*n* = 9/group). (I) Double immunofluorescence labelling of the spinal cord anterior horn at 28dpo with anti‐Chat and anti‐5HT antibodies. (J) Quantification of the 5HT positive area (*n* = 9/group). Graphical data are presented as the mean ± standard deviation. Scale bar = 50 μm. DAPI (blue) labeled cell nucleus. R: right, L: left, V: ventral, D: dorsal. *p < 0.05, **p < 0.01, ***p < 0.001

The fibrotic scar was located in the center of the lesion and surrounded by a glial scar (white curve in the Figure 2A). The fibrotic scar area was reduced in the 5‐FU‐treated group at 28dpo (GFAP‐negative area, Figure 2B). 5‐FU treatment reduced the protein levels of aggrecan and fibronectin (Figure 2C,D). Immunofluorescence labeling also showed obvious reductions of aggrecan and fibronectin expression following 5‐FU administration at 28dpo (Figure 2E–G). Data of fibroblast and glial cell accumulation in the epicenter at acute (1dpo) and subacute phase (7dpo) after SCI showed that 5‐FU treatment reduced the fibronectin^+^, CD11b^+^, and GFAP^+^ cells accumulation at the onset of the acute phase (Figure 2H,I). Similarly, macrophages (NG2^+^ cells), meningeal and/or vascular derived fibroblasts (vimentin^+^ cells), microglia/macrophages (Iba1^+^/CD11b^+^ cells), and pericytes (PDGFRβ^+^ cells) were depleted in the 5‐FU‐treated group relative to the vehicle group (Figure 3A, a1‐a4 in Figure C). Both Iba1, PDGFRβ and vimentin positive areas were decreased after 5‐FU administration at 28dpo (Figure 3D–F).

Furthermore, 4 weeks after treatment, 5‐FU increased the neurofilament (NF)^+^ fibers in the ipsilateral side 3 mm caudal to the injury site (Figure 3G,H). The positive area of serotonergic raphespinal tract (RST) axons (5‐hydroxytryptamine [5HT] positive fiber) innervating the cholinergic neuron (Chat positive cells) in the ventral horn 3 mm caudal to the lesion site (red box in Figure 3B) increased significantly in the 5‐FU treated groups compared to vehicle rats (Figure 3I,J).

### 3.4 5‐FU treatment induces apoptosis and inhibits stromal cells proliferation and migration by reducing MMP9

**FIGURE 4:**
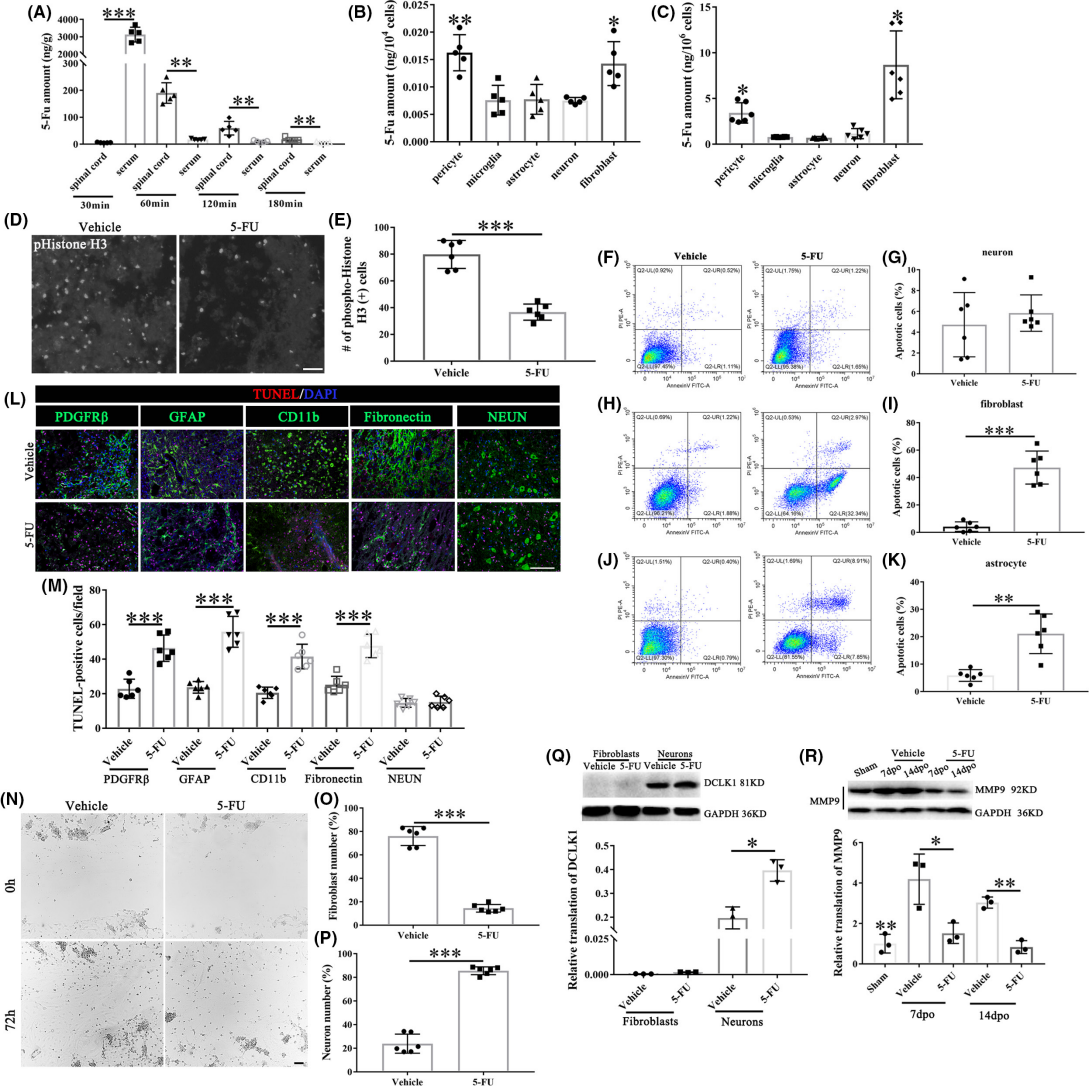
5‐FU treatment induces apoptosis and inhibits stromal cells proliferation and migration. (A) Mass spectrometric analysis of spinal cord and serum after a single 5‐FU intraperitoneal injection, *n* = 5 animals per time‐point. (B,C) 5‐FU is mainly distributed in stromal cells in vivo and in vitro (*n* = 6/group). (D) Cells labeled with the proliferation marker phospho‐Histone‐H3 after spinal cord hemisection at 28dpo. (E) Number of phospho‐Histone‐H3‐positive (+) cells at the lesion site (*n* = 6/group). (F,G) Apoptotic neurons were measured by annexin FITC/PI analysis assay co‐cultured with 5FU for 48 h (*n* = 6/group). (H,I) Apoptotic fibroblasts were measured by annexin FITC/PI analysis assay co‐cultured with 5FU for 48 h (*n* = 6/group). (J,K) Apoptotic astrocytes were measured by annexin FITC/PI analysis assay co‐cultured with 5FU for 48 h (*n* = 6/group). (L) Cell‐type‐specific TUNEL staining images of injured spinal cord section at 7dpo. Scale bar = 100 μm. (M) Quantification of the TUNEL positive cells in different cell types (*n* = 6/group). (N) 5‐FU inhibits fibroblast migration rather than neurons, into the cell‐free area, in the wound healing assay. Scale bar = 50 μm. (O,P) Number of fibroblasts and neurons in the cell‐free area after 72 h (*n* = 6/group). (Q) Expression levels of DCLK1 in fibroblasts and spinal cord neurons with different treatments were measured via western blotting (*n* = 3/group). (R) Expression levels of MMP9 in the injured spinal cord (*n* = 3/group). Graphical data are presented as the mean ± standard deviation. *p < 0.05, **p < 0.01, ***p < 0.001

Mass spectrometry confirmed that after i.p. injection in rats, 5‐FU was rapidly absorbed into the injured spinal cord and remained at comparable levels for at least 180 min (Figure 4A). And, there was no statistical difference between intact and injured spinal cord in the content of 5‐FU (see Figure S2). 5‐FU was mainly distributed in pericytes and fibroblasts in the injured spinal cord compared with glia cells and neurons (Figure 4B). Cell uptake experiment also confirmed that higher amount of 5‐FU uptake occurs in the pericytes and fibroblast in vitro (Figure 4C). 5‐FU significantly reduced the number of phospho‐Histone‐H3 (pHistone H3) (+) cells at the lesion site at 28dpo (Figure 4D,E). Flow cytometry analysis suggested that the apoptosis of fibroblasts and astroglia was accelerated after 5‐FU administration for 48 hours but with little influence on neurons in vitro (Figure 4F–K). In addition to neurons, the apoptosis of pericytes, astrocytes, microglia and fibroblasts was increased in rats i.p. injected with 5‐FU (Figure 4L,M). In co‐cultures of meningeal fibroblasts and postnatal spinal neurons, 5‐FU inhibited migration of meningeal fibroblasts (Figure 4N,O) while also enhancing the migration of neurons in wound healing assays (Figure 4N,P). The dual role of 5‐FU in primary neurons was associated with neuron‐specific expression of DCLK1, which plays an important role in neuronal migration (Figure 4Q). MMP9 expression was significantly decreased over time in the spinal cord of 5‐FU‐treated animals compared with that of the vehicle group start on the 7dpo (data of 7dpo and 14dpo only shown, Figure 4R). We also detected the expression of matrix metalloproteinase 2 (MMP2), and there was no significant difference between these two groups (see Figure S3).

**FIGURE 5:**
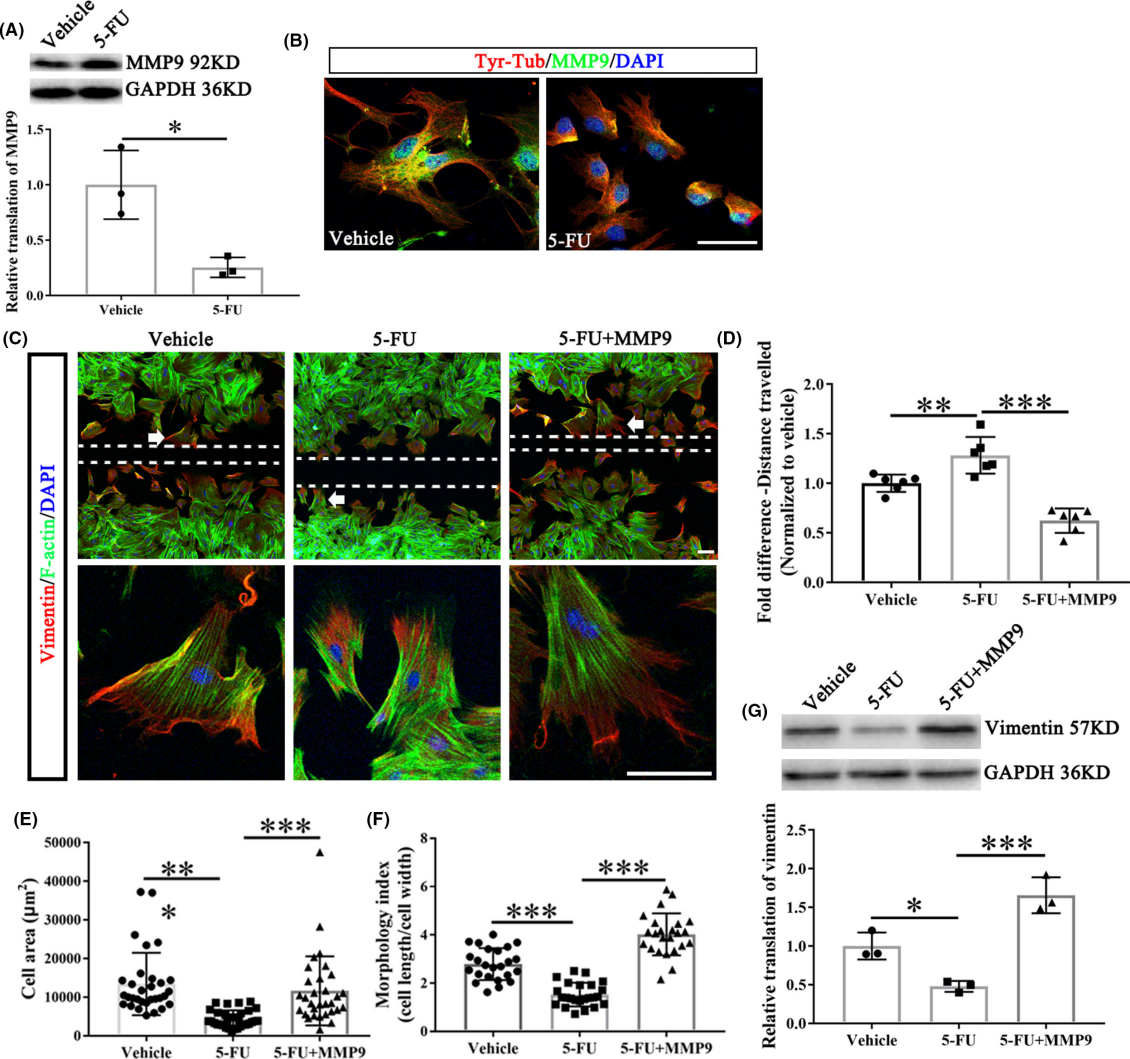
5‐Fu treatment inhibits fibroblasts migration through reducing MMP9. (A) The representative protein bands show the levels of MMP9 in the fibroblasts treated with or without 5‐FU (*n* = 3/group). (B) The meningeal fibroblasts were stained with tyrosinated tubulin (Tyr‐Tub, dynamic microtubules), MMP9 and DAPI. Scale bar = 50 μm. (C) Meningeal fibroblasts stained for vimentin, F‐Actin and DAPI in vehicle and 5‐FU groups with or without rMMP9. The bottom panel indicated the enlarged views of white arrow showing areas. Scale bar = 100 μm for all panels. (D) The size of the wound was presented as the fold change among different group (*n* = 6/group). (E) Cell area quantifications of fibroblasts near the wound center (area of white dotted line in Figure C) (*n* = 30/group). (F) Morphology index of fibroblasts with or without indicated treatments (*n* = 24/group). (G) The protein level of vimentin (*n* = 3/group). DAPI (blue) labeled cell nucleus. Graphical data are presented as the mean ± standard deviation. *p < 0.05, **p < 0.01, ***p < 0.001

The protein level of MMP9 in fibroblasts was significantly reduced after 5‐FU incubation (Figure 5A,B). Recombinant MMP9 (rMMP9) co‐incubation resulted in a significant increase in the distance traveled, cell size, and morphology index in 5‐FU treated fibroblasts (Figure 5C–F). Besides, the vimentin fibers were clustered at the edge of the cell and oriented the direction of migration in the vehicle and 5‐FU plus rMMP9 group (Figure 5C). rMMP9 also elevated the vimentin expression which was reduced by 5‐FU (Figure 5G).

### 3.5 5‐FU abrogates polarity of meningeal fibroblasts

**FIGURE 6:**
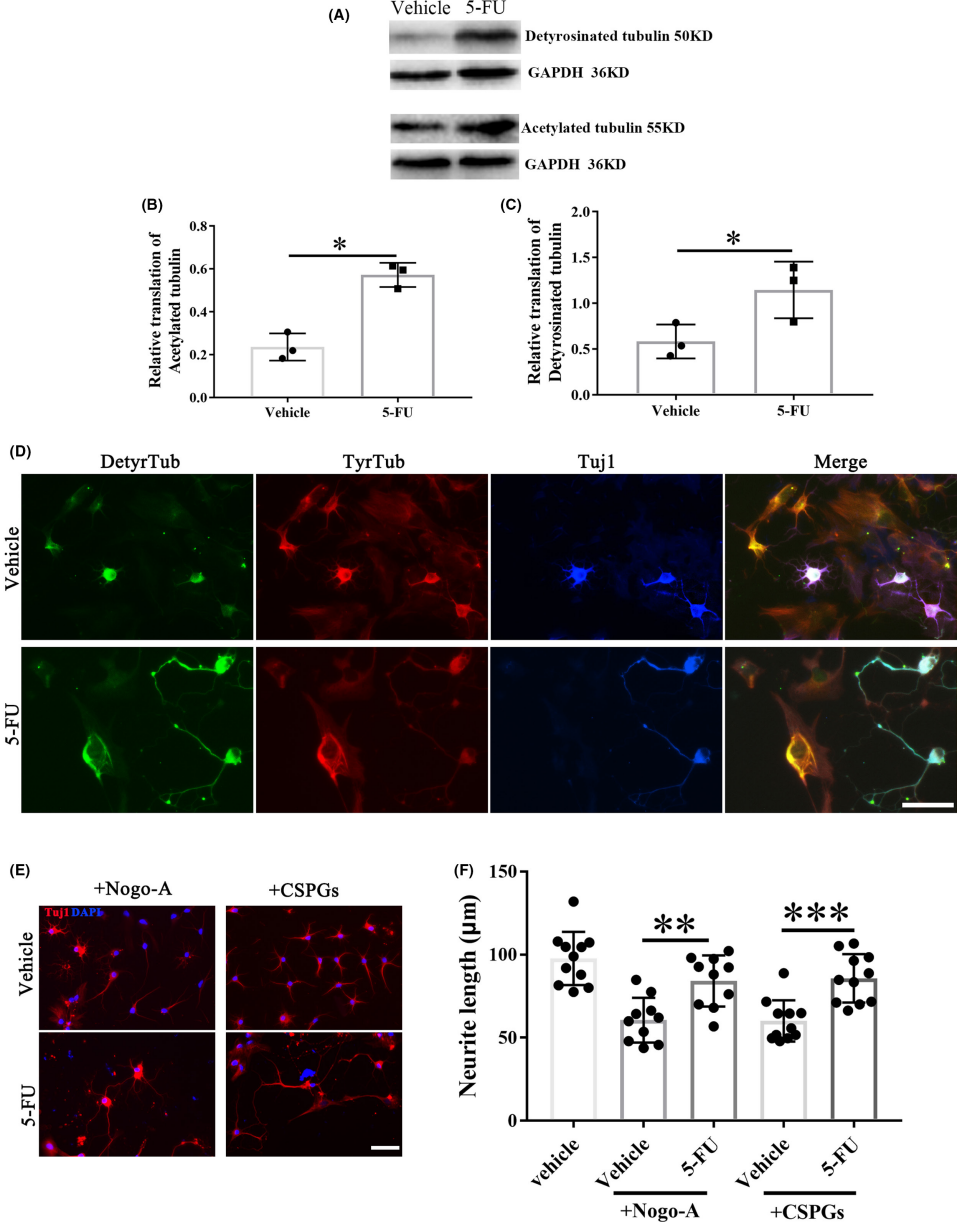
Effects of 5‐FU on microtubule stability. (A) The representative protein bands show the levels of detyrosinated and acetylated tubulin in the lesion site extracts of rats treated with vehicle or 5‐FU. (B,C) Quantification of detyrosinated tubulin and tyrosinated tubulin in vehicle and 5‐FU groups (*n* = 3/group). (D) Co‐cultures of spinal neurons and meningeal fibroblasts stained for detyrosinated tubulin (stable microtubules) and tyrosinated tubulin (dynamic microtubules). (E) Tuj‐1 immunolabeling of spinal neurons growing on inhibitory substrates (Nogo‐A and CSPGs). (F) Neurite length quantifications of spinal neurons with (+) or without (−) indicated treatments (*n* = 10/group). Scale bar = 50 μm. DAPI (blue) labeled cell nucleus. Graphical data are presented as the mean ± standard deviation. *p < 0.05, **p < 0.01, ***p < 0.001

Rats injected with 5‐FU immediately for 4 consecutive days showed increased levels of detyrosinated and acetylated tubulin in lesion site extracts 4 weeks after spinal cord hemisection (Figure 6A–C). 5‐FU abrogated the polarity of meningeal fibroblasts by elevating the levels of detyrosinated microtubules (DetyTub, green) (Figure 6D). Besides, following 5‐FU application, the neurite length in neurite outgrowth inhibitor‐A (Nogo‐A) and chondroitin sulfate proteoglycans (CSPGs) groups was all significantly increased compared to that in vehicle group (Figure 6E,F).

## 4 DISCUSSION

Here, we investigated the effect of 5‐FU on fibrotic scar formation. 5‐FU administration reduced fibrotic scarring by inhibiting MMP9 and stabilizing microtubules after SCI with no obvious kidney and hepatic dysfunction. Previous studies have reported that attenuating fibrotic scarring promotes axon regeneration and functional recovery after SCI.^5^ Earlier studies modifying the ECM associated with fibrotic scarring also provided suggestive evidence.^18^ We also evaluated the effects of 5‐FU on the expression of aggrecan and fibronectin, and of stromal cell aggregation (e.g., NG2^+^, vimentin^+^, Iba1^+^/CD11b^+^, and PDGFRβ^+^ cells). The results showed that 5‐FU administration depressed these components of fibrotic scar.

Spontaneous ipsilesional hindlimb recovery of joint articulation, active stepping, and weight support during locomotion in the open field occurred during the first 4 weeks after hemisection with residual deficits in performance remaining until the end of experimental evaluation similar to a previous report.^19^ In our study, we placed the bars irregularly (1‐3 cm spacing) and changed in every testing session to prevent habituation to a fixed bar distance.^20^, ^21^ Results showed that the ipsilesional hindlimb deficits at 3 and 4 weeks were approximately 50%–60% which were different from these data collected form models running in a fixed bar distance.^19^ Lower hind limb errors and higher BBB scores correspond to better hindlimb motor ability and better coordination function.^22^ 5‐FU systematic administration significantly increased BBB scores and reduced the hind limb errors, revealing that 5‐FU promoted the recovery of hind limb locomotor activity after hemi‐section SCI in rats. Meanwhile, the thermal and mechanical withdrawal thresholds in the 5‐FU group were significantly decreased compared to the vehicle group, indicating that 5‐FU exerts a therapeutic effect on the recovery of hind limb sensory function.

It may be argued that the enhanced recovery of motor ability and sensation is due to the improvement of the local microenvironment at the injury site.^23^ Therefore, we investigated the scar area and neuronal activity near the lesion. The Nissl body is the main component of protein synthesis in neurons.^24^ The status of neurons can be indicated by Nissl bodies as they can shrink or disappear when neurons are over‐stimulated.^25^, ^26^ Once the destruction factor disappears, the Nissl body can rebound by simultaneously restoring protein synthesis and cell metabolism.^27^, ^28^, ^29^ The 5‐FU‐treated group exhibited a smaller scar area and more obvious rebounded Nissl bodies near the scar of the gray matter after hemisection SCI.

To further assess the improvement of the local microenvironment at the injury site, we detected the expression of aggrecan, which is one of the inhibitory ECMMs. These molecules are secreted by stromal cells,^10^, ^11^ generating physical and chemical barriers, thus preventing damaged axons from passing through the injured site.^7^, ^30^ Other scarring molecules like fibronectin will aggravate scar formation and thus inhibit axonal growth.^12^, ^31^, ^32^ Instead of suppressing neuronal growth, other studies showed that fibronectin promote axon growth. Fibronectin supports neurite outgrowth and axonal regeneration of adult brain neurons.^33^, ^34^ Those evidences demonstrated that the function of fibronectin on axon growth and neural repair is controversy.^35^ In this study, our data supports the inhibitory activity of the fibronectin. 5‐FU administration significantly reduced the deposition of aggrecan and fibronectin, indicating that 5‐FU attenuates fibrotic scar formation. In recent works, prolonged microglial activation in the injury site has been linked to impaired parenchymal healing and functional restoration.^36^, ^37^ However, neonatal microglia expressing a number of peptidase inhibitors permits the growth of long projecting axons through the lesion.^37^ The presence and apoptosis numbers of microglia were quantified. 5‐FU treatment reduced the microglia accumulation at acute (1dpo) and subacute phase (7dpo) after SCI, and increased apoptosis of the microglia. In addition to fibrous scars, glial scars catch more and more researchers' attention.^38^, ^39^, ^40^ Several strategies have been described to reduce astrogliosis and scar formation for spinal cord injury recovery, such as Overexpression of the transcription factors OCT4 and KLF4,^39^ EphB2 knockdown^38^ and Feasible stabilization of chondroitinase abc.^40^ Here, we found that 5‐FU treatment reduced the fibroblast and astrocyte accumulation at acute (1dpo) and subacute phase (7dpo) after SCI, and increased apoptosis of the fibroblast and astrocyte. Those data demonstrated that the key mechanism of 5‐FU is reducing the fibronectin^+^, CD11b^+^ and GFAP^+^ cells accumulation at the onset of the acute phase.

It has been reported that a large number of stromal cells accumulate in fibrotic scars, including NG2+ macrophages (NG2^+^ cells), meningeal and/or vascular derived fibroblasts (vimentin^+^/αSMA^+^ cells), pericytes (PDGFRβ^+^ cells), and ependymal cells.^41^ 5‐FU administration significantly reduced the number of NG2^+^ cells, vimentin^+^ cells, and PDGFRβ^+^ cells, further confirming that 5‐FU inhibits fibrotic scar formation.

PDGFRβ is first secreted by platelets, and then by fibroblasts, macrophages, endothelial cells, and epidermal cells in the wound.^42^ PDGFRβ‐positive cells were pericytes that had accumulated in the lesion core of SCI. Reducing pericyte‐derived scarring promotes recovery after SCI.^5^ NF is a main component of the neuronal cytoskeleton, indicates neural sprouting.^43^ 5‐HT is closely related to the hypersensitivity reaction and the molecular biological basis for pathologic hyperreflexia and tonic spasm after SCI.^44^ 5‐FU administration significantly promoted the NF and 5‐HT positive fibers crossing the lesion area, which was the basis of sensorimotor functional recovery. The observed increase in axon density caudal to the hemisection in 5‐FU group can be a result of true regeneration of transected axons and/or sprouting of nearby axons on the contralateral that have been spared by the injury.^5^

This effect of 5‐FU resulted from increased apoptosis and reduced cell proliferation and fibroblast migration. Given the comparable central nervous system penetration,^45^ 5‐FU was rapidly absorbed and mainly distributed in pericytes and fibroblasts in the injured spinal cord. Next, we found that 5‐FU administration induced apoptosis of the stromal cells, such as pericytes, astrocytes, microglia, and fibroblasts. These results were similar to those of previous studies,^46^, ^47^ which further uncovered the key mechanism of 5‐FU on fibrotic scar formation. The MMP family is involved in tissue remodeling and angiogenesis,^48^ and the expression of MMP2 and MMP9 reflect invasion. Here, we found that 5‐FU treatment inhibited fibroblast migration by reducing MMP9. These results were similar to those of previous studies, which showed that expression of MMP2 and MMP9 was suppressed after 5‐FU treatment.^49^ Meanwhile, we found that vimentin fibers were inhibited by 5‐FU through MMP9. Vimentin have a significantly greater ability to resist stress without breaking in vitro compared with actin or microtubules and also to increase cell elasticity in vivo.^50^ Here, 5‐FU inhibited the fibroblast migration through disturbing and downregulating the vimentin fibers. Interestingly, 5‐FU plays an opposite role in neuronal migration. The dual role of 5‐FU in primary neurons was associated with neuron‐specific expression of DCLK1, which plays an important role in the migration of neurons.^51^

Our study also confirmed that 5‐FU abrogated the polarity of meningeal fibroblasts. Detyrosinated and tyrosinated microtubules are hallmarks of directed cell migration. Detyrosinated tubulin (+) represents stable microtubules, and tyrosinated tubulin (+) represents dynamic microtubules.^52^ 5‐FU‐treated fibroblasts were round and non‐polar, with increased levels of detyrosinated tubulins. Nogo‐A is the strongest nerve growth inhibitory factor,^53^ and CSPGs form scars at the lesion site,^7^ preventing the damaged axon from passing through the lesion site.^7^, ^30^ The current study showed that 5‐FU administration promotes the axon growth when Nogo‐A and CSPGs existed, which confirmed its effect on axon regeneration after hemi‐SCI.

Nevertheless, there are some several limitations in this study. First, we only recruited the female rats to establish SCI models. Because sex differences exist for CNS microstructures,^54^ metabolisms,^55^ cerebral blood flow,^56^ and injury recovery,^57^ the potential sex differences in spinal cord injury should be addressed in future investigations. Second, therapeutic effects against injury and the animals' response to 5‐fluorouracil also may be influenced by sex differences.^58^

Collectively, 5‐FU administration exerted inhibitory effects on fibrotic scar formation by preventing the proliferation and migration of stromal cells and abrogating the polarity of meningeal fibroblasts on the lesion site after hemisection‐SCI in rats, which can reduce the scar area and promote axonal regeneration.

## AUTHOR CONTRIBUTIONS

X.Y. X.H., and W.Y. performed experiments with assistance from P.X. J.J. provided reagents and input into study design. X.Y., Y.K., and T.W. conceived and designed the study. P.X. and J.J. analyzed the data and provided input into study design. W.T. supervised the study. X.Y., W.Y., and J.J. wrote the manuscript with input from all authors.

## CONFLICT OF INTEREST

The authors declare no competing interests.

## Supporting information


Supinfo

Figure S1

Figure S2

Figure S3

Appendix S1

## Data Availability

The data and material presented in this manuscript is available from the corresponding author on reasonable request.
