# Supplementary material for: 5‐Fluorouracil reduces the fibrotic scar via inhibiting matrix metalloproteinase 9 and stabilizing microtubules after spinal cord injury
Source: CNS Neurosci Ther. 2022 Aug 2;28(12):2011–23. doi: 10.1111/cns.13930 (PMC9627390; doi:10.1111/cns.13930)
Supplement: Supplementary file 1 — Supinfo [file CNS-28-2011-s004.docx]

**Full-length blots**


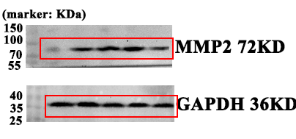


Full unedited gel/blot for Figure S1


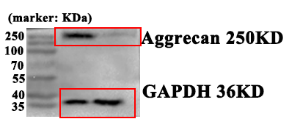


Full unedited gel/blot for Figure 2C


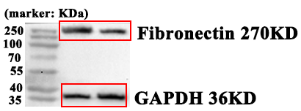


Full unedited gel/blot for Figure 2D


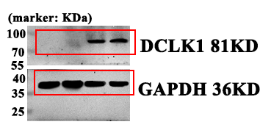


Full unedited gel/blot for Figure 4F


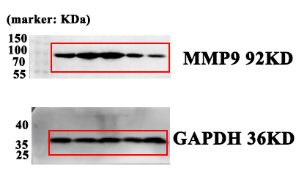


Full unedited gel/blot for Figure 4G


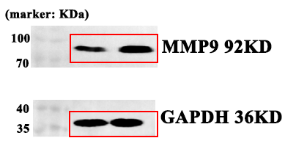


Full unedited gel/blot for Figure 5A


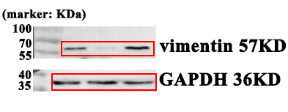


Full unedited gel/blot for Figure 5G


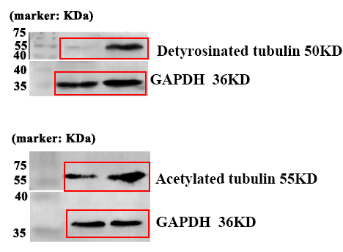


Full unedited gel/blot for Figure 6A
