# Supplementary figures and images for: 5‐Fluorouracil reduces the fibrotic scar via inhibiting matrix metalloproteinase 9 and stabilizing microtubules after spinal cord injury
Source: CNS Neurosci Ther. 2022 Aug 2;28(12):2011–23. doi: 10.1111/cns.13930 (PMC9627390; doi:10.1111/cns.13930)

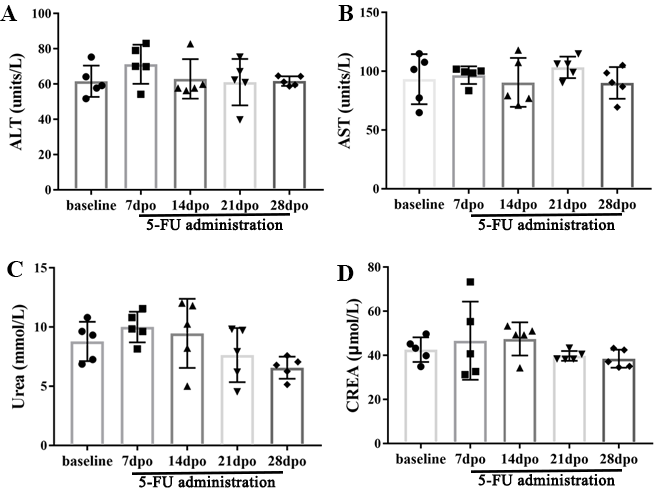

Supplement: Supplementary file 2 — Figure S1 [file CNS-28-2011-s005.tif]

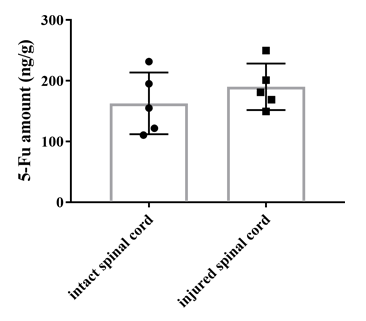

Supplement: Supplementary file 3 — Figure S2 [file CNS-28-2011-s001.tif]

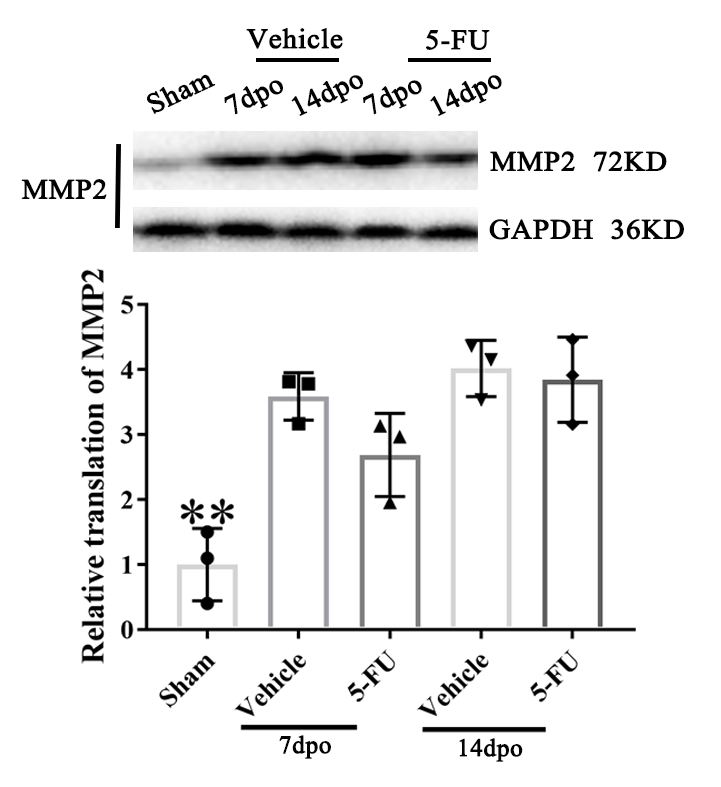

Supplement: Supplementary file 4 — Figure S3 [file CNS-28-2011-s003.tif]
