# Supplementary material for: 5‐Fluorouracil reduces the fibrotic scar via inhibiting matrix metalloproteinase 9 and stabilizing microtubules after spinal cord injury
Source: CNS Neurosci Ther. 2022 Aug 2;28(12):2011–23. doi: 10.1111/cns.13930 (PMC9627390; doi:10.1111/cns.13930)
Supplement: Supplementary file 5 — Appendix S1 [file CNS-28-2011-s002.docx]

**5-fluorouracil reduces the fibrotic scar via inhibiting matrix metalloproteinase 9 and stabilizing microtubules after spinal cord injury**

**5-fluorouracil reduces the fibrotic scar**

**Yang Xu^1,4^, Xiuying He^1,4^, Yangyang Wang^1^, Jiao Jian^2^, Xia Peng^2^****, Lie Zhou^3^,** **Yi Kang^4,5^, Tinghua Wang^1,2,5^**

**^1^** Institute of Neurological Disease, West China Hospital, Sichuan University & The Research Units of West China, Chinese Academy of Medical Sciences, Chengdu 610041, China. **^2^** Institute of Neuroscience, Laboratory Zoology Department, Kunming Medical University, Kunming 650500, China. ^3^ Yunnan Key Laboratory of Stem Cell and Regenerative Medicine, Biomedical Engineering Research Center, Kunming Medical University, Kunming 650500, China. ^4^ Laboratory of Anesthesia and Critical

Care Medicine, Department of Anesthesiology, Translational Neuroscience Center, West China Hospital, Sichuan University, Chengdu, 610041, China. ^5^ National-Local Joint Engineering Research Center of Translational Medicine of Anesthesiology, West China Hospital, Sichuan University, Chengdu, 610041, China

Yang Xu, Xiuying He are contributed equally to this work.

*Correspondence

Tinghua Wang, Institute of Neurological Disease, West China Hospital, Sichuan University & The Research Units of West China, Chinese Academy of Medical Sciences, Chengdu 610041, China. Institute of Neuroscience, Laboratory Zoology Department, Kunming Medical University, Kunming 650500, China. Email: [Wangtinghua251@163.com](mailto:Wangtinghua251@163.com)

Yi Kang, Laboratory of Anesthesia and Critical Care Medicine, Department of Anesthesiology, Translational Neuroscience Center, West China Hospital, Sichuan University, Chengdu, 610041, China. Email: [kangyi1@sina.com](mailto:kangyi1@sina.com)

**Author Contributions**X.Y. X.H. and W.Y. performed experiments with assistance from P.X.. J.J. provided reagents and input into study design. X.Y., Y.K. and T.W. conceived and designed the study. P.X. and J.J. analysed the data and provided input into study design. W.T. supervised the study. X.Y., W.Y. and J.J. wrote the manuscript with input from all authors.

**Abbreviations:** 5-FU, 5-fluorouracil; SCI, spinal cord injury; NG2/CSPG4, chondroitin sulfate proteoglycan 4; PDGFRβ, platelet-derived growth factor receptor beta; MMP9, matrix metalloproteinase 9; DCLK1; doublecortin-like kinase 1; ECMMs, extracellular matrix molecules; GFAP, glial fibrillary acidic protein; BBB, Basso Beattie Bresnahan; dpo, day post operation; i.p., intraperitoneal; WBC, white blood cell; RST, serotonergic raphespinal tract; 5HT, 5-hydroxytryptamine; CNS, central nervous system; pHistone H3, phospho-Histone-H3; DetyTub, detyrosinated microtubules; Nogo-A, neurite outgrowth inhibitor-A; CSPGs, chondroitin sulfate proteoglycans; MMP2, matrix metalloproteinase 2; LC-MS/ MS, Liquid chromatography tandem mass spectrometry; ALT, alanine aminotransferase; AST, aminotransferase; CREA, creatinine.

**Supplementary methods**

**Animal**

Adult female Sprague Dawley (SD) rats (225-250 g), provided by the Experimental Animal Center of Sichuan University, were housed under conditions of proper temperature, humidity and adequate ventilation, under a 12 h light/dark cycle, with free access to food and water. Animal feeding and care were conducted in strict compliance with the Chinese Experimental Animal Protection and Ethics Committee and guidelines for the care and use of laboratory animals published by the National Institutes of Health. This study protocol was approved by "sichuan provincial committee for experimental animal management". All procedures were carried out in compliance with ARRIVE guidelines.

**Animal model establishment**

Briefly, after being deeply anesthetized by continuous inhalation of isoflurane (2% isoflurane/oxygen), a partial laminectomy of the 10^th^ thoracic vertebrae was made to expose the spinal cord. Subsequently, thoracic (T10) spinal cord hemisection, on the right side of the spinal cord, was performed using a scalpel and micro-scissors under microscope visualization. After hemostasis and suturing, rats were maintained with their dams, until they recovered. Sham rats were subjected to surgical dissection and exposure. After surgery, rats were injected with 0.5 mg/100 g cefotaxime sodium antibiotics for 3 days. Meanwhile, the 5-FU group was exposed to intraperitoneal (i.p) injections of 5-FU (5 mg/kg) immediately for 4 consecutive days, and the same dose of normal saline was administered to the vehicle group. Bladders were manually emptied twice daily until micturition returned.

**Basso Beattie Bresnahan (BBB) scale**

After surgery, the motor function of the right side of hind limbs was evaluated using the BBB scale. Tests were conducted by three observers who did not know the experimental design, and the movements of the hindlimb ipsilateral to the injury site were scored according to the BBB grading standards. The scoring criteria were as follows: 1) 0-7 points, joint activity; 2) 8-13 points, gait and coordination function; 3) 14-21 points, claw movement. Baseline values were obtained on both sides of the hind paws. Absence of dysfunction was scored as 21 points and hind limb paralysis was scored as 0 points.

**Horizontal ladder-walking test**

Skilled locomotion, sensorimotor integration and limb placement can be evaluated using the horizontal ladder-walking test. Briefly, prior to injury, rats were pre-trained for one week on the horizontal ladder (100 cm long horizontal runway of metal bars elevated 30 cm from the ground) and assessed on the day before the surgery, to summarize the baseline data. Here, we placed the bars irregularly (1-3 cm spacing) and changed in every testing session to prevent habituation to a fixed bar distance. After spinal cord hemisection, rats were tested weekly for four consecutive weekends. Experiments for each rat were performed in triplicate. The average number of misplaced hind limb movements, while the rat run along the horizontal ladder in constant motion from start to end, were manually recorded by an observer blinded to the design. The percentage of misplacements for the hindlimb ipsilateral to the injury site was analyzed by averaging across the three trials for each rat.

**Thermal hyperalgesia test**

The thermal hyperalgesia test, called the Hargreaves’ Planter Test, was performed with a standard apparatus. The targeted hind limbs of rats were given a thermal radiant stimulus and the latency of the paw withdrawal response was measured automatically. Baseline values were obtained on both sides of the hind paws. The stimulation was automatically ended at 20 s in refractory rats, to prevent tissue damage. The paw withdrawal latency of the injured side was performed and repeated for three consecutive times. In this experiments, three examiner were unaware of the treatment that each animal received.

**Mechanical hyperalgesia test**

The mechanical hyperalgesia test was also performed as previously described, to demonstrate changes in pain behaviors elicited by a mechanical pressure apparatus (Ugo-Basile SRL, Monvalle, Italy). Briefly, a mechanical pressure apparatus was used to apply an increasing force (measured in grams) to the plantar hind paw until a withdrawal reflex was precipitated. The mechanical paw pressure thresholds (PPT) were determined weekly, in all of the groups. Baseline values were obtained on both sides of the hind paws. PPT measurements were assessed on the right side for three consecutive times. The final PPT was calculated as the mean obtained from all rats in each group. In this experiments, the examiner was unaware of the treatment that each animal received.

**Sample harvest**

Animals were deeply anesthetized and transcardially perfused with 0.9% normal saline. Following 4% paraformaldehyde for immobilization, spinal cords were harvested and subsequently post-fixed in 4% paraformaldehyde for 48 h. Five-millimeter blocks of the spinal cord including injury epicenters were paraffin-embedded to make sagittal sections for examining the fibrotic scar area. Two-millimeter blocks of the spinal cord 1mm caudal to the epicenter were paraffin-embedded to make cross sections for Nissl staining. Two-millimeter blocks of the spinal cord 3-4 mm caudal to the injury site were paraffin-embedded to make cross sections for NF and 5HT immunostaining assay (Figure 3B). For western blots and liquid chromatography tandem mass spectrometry (LC-MS/ MS), the sham rats at 14 days post-operation, intact negative controls (total N=8, n=4 for each time points) and 5-FU treated rats (total N=8, n=4 for each time points), were perfused with 4˚C, 0.9% normal saline and the lesioned sections of the spinal cord removed promptly and were stored at -80˚C until assayed to detect the protein levels of MMP2/9, detyrosinated tubulin and acetylated tubulin.

**Total WBC counts**

Blood sample (1 ml) was prepared by cardiac puncture of rats immediately after opening the chest, then transferred into a tube containing anticoagulant ethylene diamine tetraacetic acid (EDTA) and kept in room temperature for measuring the total WBC counts. In this step, 0.5 ml of anticoagulant venous blood in duplicate were detected and total WBC was counted using a Neubauer hemocytometer (Burker chamber).

**Biochemical analysis**

Blood samples were collected from each rat via cardiac puncture at the end of each timepoints under anesthesia, into uncoated, non-siliconized glass bottles for biochemical analysis. Blood was allowed to clot at room temperature and centrifuged at 800 rmp for 10 min to obtain the serum. Serum activity of alanine aminotransferase (ALT) and aminotransferase (AST) as markers of hepatic function, along with creatinine (CREA) and Urea as markers of kidney function, were measured by using commercially available Mindray diagnostic kits and the chemistry analyzer system (Mindray, BS120, Shenzhen Mindray Bio-Medical Electronics Co., Ltd.).

**Flow cytometry**

Cells were obtained freshly from the spinal cord of SCI models 1h after 5-FU administration. The part of scar area was collected about 2 cm then lysed with collagenase I (1 mg/ml), collagenase II (1 mg/ml), collagenase IV (1 mg/ml) and DNase (20 U/ml) for 30 min after dissected the meninges from the tissues and cut into pieces. The remaining cells were resuspended with 10% BSA and incubated on slow rotation with Rat Fc Block (Rat CD32 Pure D34-485; BD Pharmingen) for 5 min at room temperature to prevent nonspecific binding. Then, cells were incubated with PDGFRB Polyclonal Rabbit Antibody (Proteintech), a specific marker of pericyte, on slow rotation at 4℃ for 30 min, and the following antibodies (R-PE conjugated Goat Anti-Rabbit IgG(H+L) (Proteintech); GFAP Monoclonal Antibody (GA5), Alexa Fluor 488, eBioscience™ (Invitrogen), a specific marker of astrocyte; CD11b/c Monoclonal Antibody (OX-42), APC (Invitrogen), a specific marker of microglia; CD271 (NGF Receptor) Monoclonal Antibody (ME20.4), Super Bright 600, eBioscience™(Invitrogen), a specific marker of neuron; Alexa Fluor® 750 Mouse Anti-Human Vimentin (BD Pharmingen), a specific marker of fibroblast) were used to incubated cells at 4℃ for 30 min after cells were washed twice. After incubation, cells were washed and resuspended with 3% BSA for flow cytometry analysis and FACS-based isolation using flow cytometer (BD Biosciences, FACSAria SORP, America). Five different isolated population of cells will be obtained. 100,000 single live events per isolated clusters were used to detected the 5FU amounts by LC-MS/ MS.

**LC-MS/MS**

Tissues and cells were rinsed twice and homogenized with normal saline. Sample lysates were spun down at 12,000 g, at 4 ℃ for 5 min to collect the supernatant. The LC–MS/MS analysis was performed using an Agilent 6460 triple quadrupole mass spectrometer equipped with an electrospray ionization source (Agilent Technologies, CA, USA). Samples were separated by a Waters Xbridge Amide column (100 mm × 3 mm, 3.5 μm) column with 5-bromouracil as the internal standard. The total running time for one injection was 6 min, and the temperature of column was set as 35 °C. Mass spectrometry conditions were optimized for maximal sensitivity in a negative ionization mode, which was set up as follows: the sheath gas flow rate of 11.0 l/min; sheath gas heater temperature of 300°C; nebulizer pressure at 45 psi; capillary voltage at 3,500 V. The following multiple-reaction monitoring transitions were monitored: m/z 128.9→42 for 5-Fu and m/z 188.8→42 for an internal standard (5-Br, IS). Data were analyzed using the MassHunter software (B.04.00 Build 4.0.479.0, Agilent Technologies).

**Nissl staining**

Paraffin-embedded tissues were sectioned and deparaffinized, and then stained with a 1% cresyl violet (Sigma) solution, in a 60℃ incubator for 30 s. Subsequently, 95% ethanol was added for swift differentiation until the Nissl bodies were purple and the surrounding tissues were colorless. Finally, the specimens were dehydrated using absolute ethyl alcohol, transparentized by xylene and sealed with neutral gum. Images were taken using a light microscope.

**Cell culture**

Primary spinal neurons were harvested from the spinal cord of newborn SD rats. Briefly, tissues were cut and isolated with trypsin 0.25%. Following centrifugation (5 min, 800 rpm, at room temperature), the supernatant was discarded and the pallets were resuspended in complete culture medium (Hyclone) containing Dulbecco's modified eagle medium (DMEM) /high glucose, 10% fetal calf serum, and 1% penicillin-streptomycin solution. Cells were subsequently plated onto the 24-well plates and culture flasks with 500 μl/well at a density of 5×10^5^ cells/mL. Four hours later, the medium was replaced with neurobasal medium plus 2% B27. 3 days later, the culture medium was replaced with fresh complete neurobasal medium with vehicle (DMSO), 5-FU (25 μg/ml) and Nogo-A (400 ng/ml, R&D systems) or chondroitin-sulfate-proteoglycans (CSPGs, 1 µg/ml; Millipore). 48 h after treatment, cells were fixed with 4% PFA, permeabilized with PBS-Triton 0.3%, blocked with PBS-goat serum 5% and immunostained with beta-3 tubulin (Tuj1). Neurons treated with DMSO or 5-FU were lysed and immunobloted to detect the DCLK1.

Primary meningeal fibroblasts were obtained from the spinal cord of newborn SD rats. Briefly, meninges were peeled off and homogenated with 0.25% trypsin in a 37 °C incubator for 10min. Then, cells were centrifuged, resuspended and plated onto the 24-well plates and culture flasks with DMSO or 5-FU (25 μg/ml). 48 h after plating, cells were lysed and immunobloted as mentioned above.

For co-cultures of newborn rat spinal neurons and meningeal fibroblasts, the spinal cord of newborn SD rats were harvested together with the meninges. After been homogenated with trypsin, mixed cell suspension were plated onto glass bottom dishes (NEST) containing complete neurobasal (NB) medium. 3 days later, the culture medium was replaced with fresh complete neurobasal medium complemented with DMSO and 5-FU (25 μg/ml). 48 h after treatment, cells were fixed and stained with detyrosinated tubulin and tyrosinated tubulin.

Primary astrocytes were obtained from the spinal cord of newborn SD rats. Spinal cord tissues were collected from newborn rat under a sterile hood and immediately placed in ice-cold DMEM. The fine forceps were used to carefully dissect the meninges from the tissues by pulling. And then, the tissues were minced in 1 mm^3^ fragments, and digested in trypsin 0.25% and collagenase 2 mg/ml for 10 min at 37 °C. Cells were dissected by pipetting, filtered, and plated on the culture flasks in DMEM supplemented with complete culture medium. After 7 to 8 days, when astrocytes were confluent, shake the culture flask at 220 rpm for 180 min on a series orbital shaker to remove microglia and oligodendrocyte precursor cells. Cells were cultured for 3–5 passages and used as mentioned above.

**Apoptosis**

Apoptosis of cells was measured using an annexin V/PI apoptosis detection kit (Vazyme, A211) according to the supplier’s manual in vitro. Primary rat cortex and spinal neuronal cells, fibroblasts and astrocytes were seeded in six-well plates for 7 days and every three days changed half culture. The cells were given 5-FU (25 ug/ml) in culture and incubated for 48h, then collected after being digested with trypsin solution (0.25%, Cytiva). The cells were washed with cold PBS for two times and suspended in 100µl binding buffer and immunostained with AnnexinV-FITC (5μl) and PI (5μl) for 10min at room temperature in dark. Cell apoptosis was measured by using flow cytometry (Cytoflex, Beckman, China) within an hour. Data were analyzed by using FlowJo™ software (Biosciences).

The colorimetric Terminal deoxynucleotidyl transferase-mediated dUTP-biotin nick end labeling (TUNEL) assay kit (Servicebio, China) was used to evaluate the apoptosis level in the injured spinal cord according to the supplier’s manual.

**Wound healing assay**

3 days after co-cultures of spinal neurons and meningeal fibroblasts, scratches are made on cell monolayers using a pipette tip, and then the culture medium was replaced with fresh complete neurobasal medium complemented with DMSO and 5-FU (25 μg/ml). 48h after treatment, 5 different randomly selected views of each group (n=3) were captured, and images were analyzed using Image J software and the migration index is calculated. Recombinant protein (rMMP9)-based scratch assays were incubated with rMMP9 (50 ng/ml) and 5-FU (25 μg/ml) for 24 h. The same conditions for assays were performed in triplicate.

**Immunofluorescence assay**

Sections were washed three times for 5 min each, at room temperature in 0.01 M phosphate buffer saline (PBS) and blocked in PBS containing 5% goat serum and 0.2% Triton X-100, for 30 min. Sections were then incubation overnight at 4 ℃ with primary antibody (anti-aggrecan, #13880-1-AP, 1:100 dilution, Proteintech; anti-fibrinectin, ab199056, 1:100 dilution, Abcam; anti-vimentin, ab92547, 1:200 dilution, Abcam; anti-Iba1, ab178846, 1:400 dilution, Abcam; anti-CD11b, ab52478, 1:200 dilution, Abcam; anti-GFAP, ab7260, 1:200 dilution, Abcam; anti-NG2, ab139406, 1:50 dilution, Abcam; anti-PDGFRβ, CST#3169, 1:100 dilution, Cell Signaling Technology; anti-NF, ab223343, 1:400 dilution, Abcam; anti-Chat, A-AF1235a, 1:200 dilution, Abgent; anti-5HT, ab66047, 1:200 dilution, Abcam; anti-NEUN, ab177487, 1:100 dilution, Abcam; anti-pHistone H3, #9706, 1:1400 dilution, Cell Signaling Technology; anti-MMP9, ab96003, 1:100 dilution, Abcam; anti-detyrosinated tubulin, AB3201, 1:400 dilution, Millipore; anti-tyrosinated tubulin, MAB1864, 1:200 dilution, Millipore; anti-beta-3 Tubulin (Tuj1), MA1-19187, 1:500 dilution, Invitrogen). The next day, sections were washed three times with PBS, for five min each, and then incubated for 1 h at 37 ℃ with a secondary antibody (goat anti-mouse IgG, Cy3, #115-165-164,1:400 dilution, Jackson; goat anti-rabbit IgG, Alexa Fluor® 488, ab150077, 1:400, Abcam). Cell nuclei were labeled with diamidine phenyl indole (DAPI). Finally, images were captured using a fluorescence microscope (Leica AF6000). All protocols have been verified with no-primary controls. ImageJ software was used to process the data and images of the immunofluorescence assay. Neurite length quantification depicted the longest neurite length of each group.

**Western blots**

Briefly, cells and spinal cord tissues were lysed with radioimmunoprecipitation (RIPA) lysis buffer containing protease inhibitor cocktail. After extraction, the samples were sonicated and centrifuged. Protein concentrations were determined using the BCA assay. After the addition of SDS-PAGE sample loading buffer, samples were heated at 90 ℃ for 5 minutes. Proteins (25 ug per lane) were electrophoresed by SDS-PAGE and then electro-transferred onto polyvinylidene fluoride (PVDF) membranes. The membranes were blocked with tris buffered saline (TBS) containing 5% milk powder (room temperature for 1.5 h). The membranes were then incubated overnight at 4 °C with primary antibodies (anti-aggrecan, #13880-1-AP, 1:1000 dilution, Proteintech; anti-fibrinectin, ab199056, 1:1000 dilution, Abcam; anti-vimentin, ab92547, 1:1000 dilution, Abcam; anti-DCLK1, A-AP20304b, 1:1000 dilution, Abgent; anti-MMP2, ab92536, 1:1000 dilution, Abcam; anti-MMP9, ab96003, 1:1000 dilution, Abcam; anti-detyrosinated tubulin, AB3201, 1:1000 dilution, Millipore; anti-acetylated tubulin, T7451, 1:1000 dilution, Sigma; anti-GAPDH, ab8245, 1:1000 dilution, Abcam). Then, membranes were washed three times and incubated for 1h with a secondary antibody (HRP-conjugated goat anti-rabbit IgG, ab6721, 1:5000 dilution, Abcam; HRP-conjugated goat anti-mouse IgG, ab6789, 1:5000 dilution, Abcam). After incubation specific bands were detected with a ChemiDoc MP System and analyzed by ImageJ software. All results were repeated for three times.
**Statistics Analysis**

All data were analyzed using SPSS 21.0 software (IBM Corporation, Armonk, NY, USA). The Shapiro-Wilk's test was applied to confirm the normality of the data. All analyses were performed using Student’s t test (two group data) or one-way analysis of variance (ANOVA) (for more than 2 group data) followed by Bonferroni's post hoc test if the data were normally distributed. While non-normally distributed variables were compared by the Mann-Whitney test (two group data) or Kruskal-Wallis test (for more than 2 group data). Statistical significance is denoted by *P < 0.05, **P < 0.01 and ***P<0.001. Data were plotted using GraphPad Prism 6.01 (GraphPad Software, CA, USA). Results were shown as means ± standard deviation.

**Supplementary Figure 1**


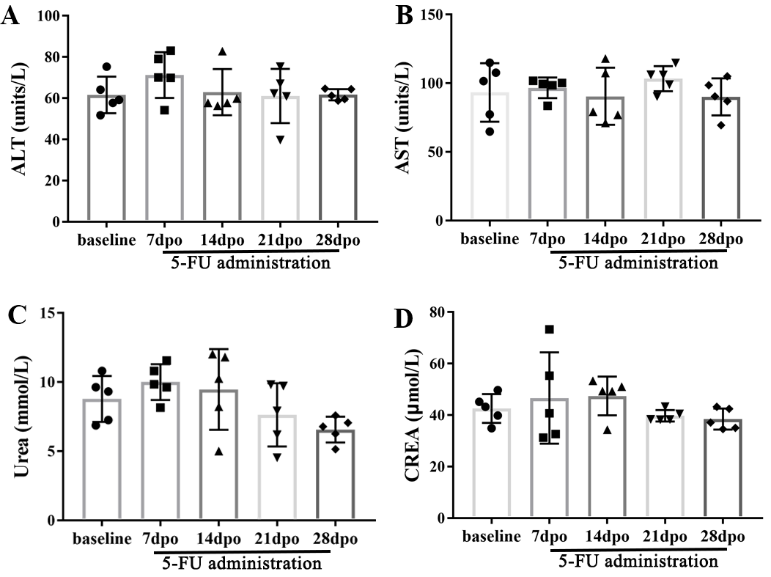


**Supplementary Fig. S1.** **There was no obvious kidney and hepatic dysfunction after 5-FU administration.** (A-B) Levels of liver function markers ALT and AST in the serum of 5-FU treated group at different time points (n=5/group). (C-D) Levels of kidney function markers Urea and CREA in the serum of 5-FU treated group at different time points (n=5/group).

**Supplementary Figure 2**


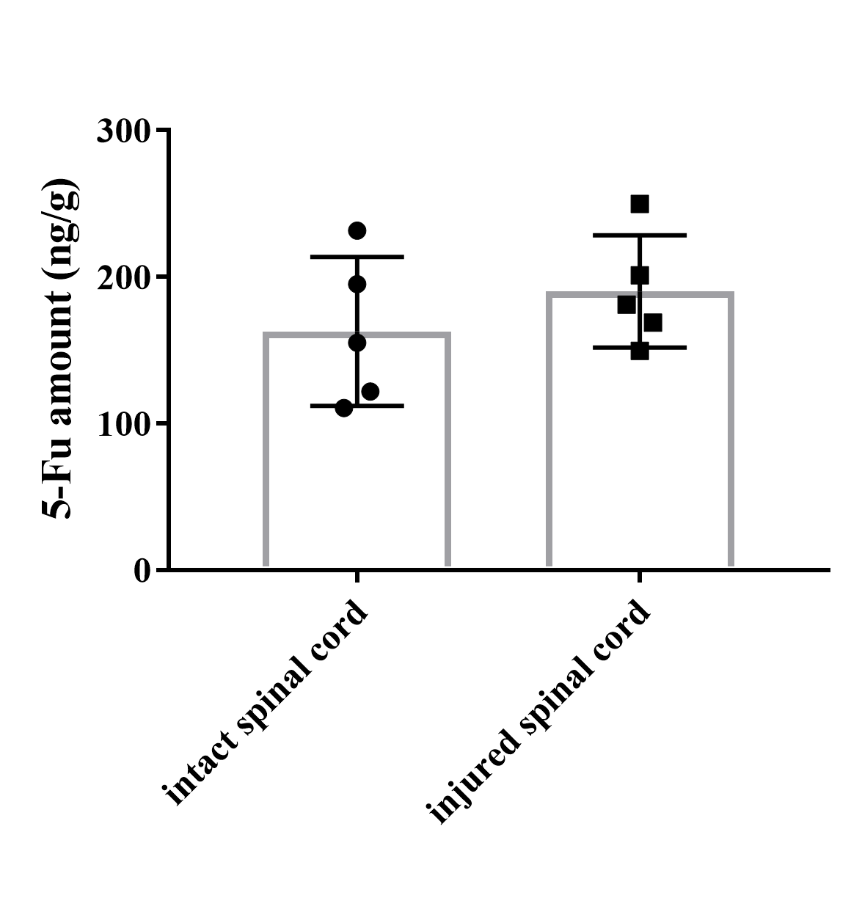


**Supplementary Fig. S2.** **There was no statistical difference of 5-FU amounts between intact and injured spinal cord.** Mass spectrometric analysis of intact and injured spinal cord 60mins after a single 5-FU intraperitoneal injection (n=5/group). Graphical data are presented as the mean ± standard deviation.

**Supplementary Figure 3**


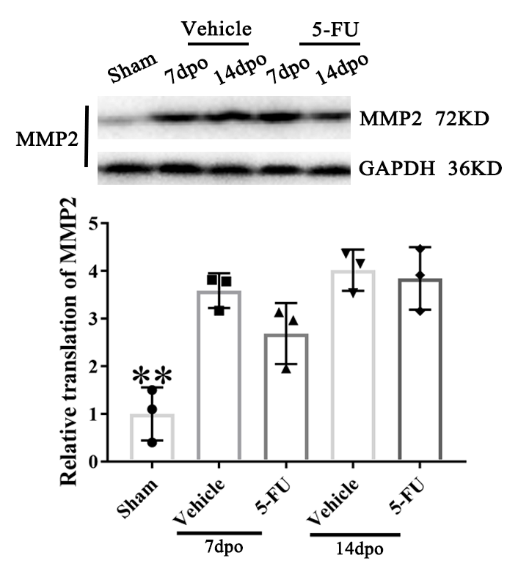


**Supplementary Fig. S3.** **5-FU administration did not affect the protein level of MMP2.** The cropped blots were displayed for MMP2 and GAPDH. Expression levels of MMP2 in the injured spinal cord (n=3/group). Graphical data are presented as the mean ± standard deviation. ***p*<0.01.
